# Benefits of Essential Oil-Enriched Chitosan on Beef: From Appearance and Odour Improvement to Protection Against Blowfly Oviposition

**DOI:** 10.3390/foods14050897

**Published:** 2025-03-06

**Authors:** Priscilla Farina, Monica Tognocchi, Giuseppe Conte, Laura Casarosa, Francesca Trusendi, Barbara Conti

**Affiliations:** 1 Department of Agriculture, Food and Environment, University of Pisa, Via del Borghetto 80, 56124 Pisa, Italy; priscilla.farina@agr.unipi.it (P.F.); monica.tognocchi@agr.unipi.it (M.T.); laura.casarosa@unipi.it (L.C.); f.trusendi@studenti.unipi.it (F.T.); barbara.conti@unipi.it (B.C.); 2 Research Center Nutraceuticals and Food for Health (Nutrafood), University of Pisa, Via del Borghetto 80, 56124 Pisa, Italy; 3 Centre for Climatic Change Impact, University of Pisa, Via del Borghetto 80, 56124 Pisa, Italy

**Keywords:** *Calliphora vomitoria*, Calliphoridae, CIE, *Laurus nobilis*, *Piper nigrum*, meat, oviposition deterrence, TBARS, VOCs

## Abstract

The food industry is increasingly turning to healthy and eco-friendly alternatives for meat preservation, with recent attention focused on chitosan (CH) and essential oils (EOs). Here, we propose two liquid formulations of CH enriched with *Laurus nobilis* or *Piper nigrum* EOs to preserve beef patties stored for 4 days at 4 °C from colour changes, secondary lipid oxidation, and alteration in volatile organic compound emissions while also preventing oviposition by *Calliphora vomitoria* on beef loaves hung for the same time at around 13 °C in a netted polytunnel. Overall, the *L. nobilis* EO-enriched CH solution increased the meat colour lightness compared to the control (+7.58%), kept redness and yellowness comparable to the control, maintained the level of thiobarbituric acid-reacting substances below the threshold for rancidity perception for at least 96 h, reduced the release of ethanol, enhanced the perception of fatty and woody notes in the meat along with the fresh, green, and citrusy aromas specific to the EO, and also provided significant protection (88.83%) against blowfly oviposition compared to the control. Therefore, the development of a spray CH formulation containing the *L. nobilis* EO appears to be a promising tool for stable and prolonged meat protection.

## 1. Introduction

According to the latest report by the Organization for Economic Co-operation and Development and the Food and Agriculture Organization [1], global meat production in 2023 was estimated at 354 million tonnes. This value is expected to reach 377 Mt by 2031 thanks to the improvements in animal breeding, management, and technologies in low- and middle-income countries, with growth in the production of beef (8%), poultry (16%), sheep (16%), and pork (17%) meat. Unfortunately, it is also estimated that 23% of meat products are lost or wasted each year, mainly at the consumption (64%), processing and production (20%), and distribution (12%) levels [2]. The causes of such spoilage are attributable to several abiotic and biotic factors occurring either independently or in combination.

For instance, meat preservation is compromised by abiotic factors like the oxidation of lipids, pigments, proteins, and vitamins, which can lead to sensory degradation (in terms of colour, texture, smell, and flavour), nutritional losses, and the potential formation of toxic substances. The initiation of oxidative processes is triggered by improper temperatures, light, oxygen, and metals as catalysts [3]. Therefore, maintaining optimal storage conditions and ensuring that the cold chain is preserved during the production, packaging, transportation, distribution, and home storage of meat products is crucial [4].

Among the biotic causes of spoilage, the primary and most relevant is the microbial growth of specific microorganisms including bacteria, fungi, and yeasts [5]. Furthermore, gravid blowfly females (Diptera: Calliphoridae) can target raw, cured, dried, cooked, and processed meat and fish for oviposition [6]. This inconvenience can happen when good manufacturing practices are not followed, meaning when the hygienic conditions of personnel, machinery, and surfaces are poor, mesh screens and traps are absent, wastes are not properly managed, and refrigeration is inadequate [7]. In nature, blowflies feed and breed on carcasses and decomposing organic matter [8], as well as on living animals and humans, causing myiasis [9]. This behaviour exposes the blowflies to several pathogenic bacteria, cestodes, and protozoa [10,11], which can subsequently be passively spread. As these “filth flies” are synanthropic insects that live in complete (eusynanthropy) or partial (hemisynanthropy) association with human settlements [12], they can also contaminate food and surfaces used for its preparation and consumption with a wide range of microorganisms, contributing to the spread of foodborne diseases.

The food industry has long sought to reduce meat loss and waste using polyvinylidene chloride (PVC) wrapping films, which are, however, highly polluting starting from their production to their hard disposal [13]. Nowadays, consumers more and more frequently request healthy, safe, and eco-friendly alternatives. Among the natural compounds already successfully employed to enhance meat preservation are chitosan (CH) and essential oils (EOs). Chitosan is an aminopolysaccharide obtained through deacetylation from chitin and is valued for its innate film-forming, antioxidant, and antimicrobial properties [14]. Essential oils are a group of heterogeneous mixtures of organic, highly volatile molecules derived from thousands of aromatic plants, including culinary herbs and spices [15], thus possessing flavouring properties [16]. Their numerous chemical constituents can act individually or synergistically as antioxidants, antiradicals, and antimicrobials, making them useful in food processing [17]. Both CH and many EOs are listed as Generally Recognised As Safe substances for consumption in the Code of Federal Regulations of the United States Food and Drug Administration. Liquid, film, and nanoparticle formulations of CH and EOs have been proposed as antioxidants [14], antimicrobials [18], and more recently, to manage insect pests [19].

Based on the evidence reported in a previous study by Farina et al. [20] regarding the protection provided by *Laurus nobilis* L. (Lauraceae) and *Piper nigrum* L. (Piperaceae) EOs formulated in a CH solution against meat dehydration, oxidative reactions, and oviposition by *Calliphora vomitoria* (Linnaeus) (Diptera: Calliphoridae) under laboratory conditions, in this work, we propose similar treatments with a double aim: (i) preserving beef patties stored for 4 days at 4 °C from colour changes, secondary lipid oxidation, and alteration in volatile organic compounds’ (VOCs) emissions and (ii) deterring *C. vomitoria* oviposition on beef loaves hung for 4 days at around 13 °C in a netted polytunnel to simulate a work environment where the good manufacturing practices are not followed. The blue bottle fly *C. vomitoria*, a species distributed across North America, Europe, and parts of Asia [21], is a key pest for the meat industry. The *L. nobilis* and *P. nigrum* EOs have already been selected by a group of expert sensory analysts for their favourable odour compatibility with meat [20], but their formulation within a stabilising matrix such as CH warrants further analysis to assess the effective benefits generated.

## 2. Materials and Methods

### 2.1. Essential Oils Purchase

The *L. nobilis* EO was purchased from Fitomedical s.r.l. (Binasco, Italy) and the *P. nigrum* EO from Sigma-Aldrich (St. Louis, MO, USA). The chemical composition of both EOs, obtained through gas chromatography/electron impact-mass spectrometry, is reported in full in Farina et al. [20].

### 2.2. Chitosan and Essential Oil-Enriched Chitosan Solutions Preparation

The CH and EO-enriched CH solutions were prepared according to the original protocol created by Peng and Li [22], slightly modified by Parichanon et al. [23]. To prepare the plain CH solution, we dispersed 1.0% (*w*/*v*) of highly viscous CH from crab shells (CAS-No. 9012-76-4, Sigma-Aldrich product No. 48165, molecular weight 500 to 700 kDa) in demineralised water containing 1.0% (*v*/*v*) of lactic acid. The solution was then stirred on a hot plate stirrer (New type are, VELP Scientifica, Usmate, Italy) at 25 °C and 250 rpm until limpid. To prepare the EO-enriched CH solutions, we added 0.50% (*v*/*v*) of vegetal glycerol, 0.60% (*v*/*v*) of Tween^®^ 80, and 1.0% (*v*/*v*) of the *L. nobilis* or *P. nigrum* EO to the plain 1.0% CH solution previously obtained. Then, we stirred the enriched solutions on the hot plate stirrer at 18 °C and 500 rpm for about 20 min. All the solutions were stored at 4 °C for no more than 7 days and heated to room temperature before use.

### 2.3. Preparation and Treatment of Beef Patties

Fresh minced beef was purchased from the Italian retailer Coop Italia s.c. in Pisa, Italy. To prepare the beef patties, approximately 100 g (99.90 ± 0.19 g; mean weight ± standard deviation (SD), *n* = 36) of meat was weighed on a precision balance (Kern 440-35A, Kern & Sohn GmbH, Balingen, Germany) to make each beef patty and manually pressed in a burger mould (diameter 8.5 cm, height 2.0 cm, surface ≅ 167 cm^2^). As the subsequent analyses performed on the patties were destructive, at time 0 (T0), we made a total of 36 beef patties (three sets of three replicates for each of the four treatments). The nine untreated (control) patties were left 60 min at room temperature (in order to equalise the processing conditions of both uncoated and coated samples), individually placed on a cellulose pulp plate (diameter 10.0 cm, height 3.5 cm), covered with PVC wrapping film, avoiding contact with the meat, and stored at 4 °C. The treated patties were first immersed for 30 s in their respective treatment (nine in the 1.0% CH solution, nine in the *L. nobilis* EO-enriched CH solution, and nine in the *P. nigrum* EO-enriched CH solution), placed on a plastic mesh grill (hole size 1.0 cm × 1.0 cm) to allow the excess solution to drip off, and left to dry for 60 min. Using the precision balance, we calculated that the CH or CH+EO covering weight was 3.82 ± 0.17 g (mean weight ± SD, *n* = 27). Therefore, we can assume that the amount of EO was 0.23 µL EO cm^−2^ of surface. Finally, the treated patties were individually placed on the pulp plates, covered with PVC film, and stored in the same refrigerator. At T0, we processed three replicates for each treatment (control, CH, *L. nobilis* EO-enriched CH, and *P. nigrum* EO-enriched CH), and then we repeated the procedure at T1 (after 48 h) and T2 (after 96 h).

### 2.4. Proximate Composition of Beef Patties

The moisture content of beef patties was determined by weight difference. We placed the samples into a ventilated oven at 105 °C for 24 h. Crude fat was measured by extracting 0.5 g of each sample with petroleum ether using an XT10 Ankom apparatus (Macedon, NY, USA) without acid hydrolysis, according to the AOCS official procedure AM 5-04. Finally, the protein content was calculated by the Kjeldahl methods according to the AOAC official procedure AOAC 981.10. The mineral level by weight difference was assessed by placing samples into a high temperature muffle furnace in which the temperature was maintained at 550 °C. All the parameters were quantified at T0, T1, and T2, as seen in Conte et al. [24].

### 2.5. Evaluation of Colour on Beef Patties

The patties were placed on a standard white tile before measurements. Colour readings were taken at four randomly selected locations on the upper surface of each circular patty to obtain a representative mean value (*n* = 4). The colour determination was performed at T0, T1, and T2. Colours were measured in the CIE (Commission Internationale de l’Éclairage) L* a* b* space [25] with an area diameter of 8 mm, including the specular component, 0% UV, D65 standard illuminant, observer angle 10°, and zero and white calibration. For measurements, we used a Minolta CM 2006d spectrophotometer (Konica Minolta Holdings, Inc., Osaka, Japan). Colour lightness (L*), redness (a*), and yellowness (b*) were recorded, whereas the hue angle (H*) and chroma (C*) indexes were calculated as follows [26]:

H* = tan^−1^ (b*/a*) expressed in degrees

C* = (a*^2^ + b*^2^)^0.5^.

### 2.6. Evaluation of Secondary Oxidation Products in Beef Patties

The secondary oxidation products of fatty acids in patties were evaluated through the thiobarbituric acid-reacting substances (TBARS) test, extracting malonyldialdehyde (MDA) with a 5% solution of trichloroacetic acid in water, as seen in Serra et al. [27]. Samples (1 g) were mixed with a 40 mM solution of thiobarbituric acid (TBA) in water and heated at 93 °C for 20 min. MDA content was determined using a spectrophotometer (Cary 50, Varian, Palo Alto, CA, USA) at a wavelength of 532 nm. TBARS were quantified by comparing the absorbance with a calibration curve obtained using a solution of tetraethoxypropane [28]. The results were expressed as mmol of MDA equivalent kg^−1^ meat [24]. The TBARS quantification was performed at T0, T1, and T2.

### 2.7. Solid Phase Microextraction-Gas Chromatography/Mass Spectrometry (SPME-GC/MS) Analysis of Beef Patties

The VOCs emitted by the beef patties were determined by the solid phase microextraction-gas chromatography/mass spectrometry technique (SPME-GC/MS), according to Serra et al. [29], with some modifications. Samples (200 μg) were placed in a 20-mL glass vial, which was sealed with an aluminium cap equipped with a PTFE-septum. The VOC mixture was collected using a stable Flex SPME fibre (75 μm; 2 cm long, DVB/Carboxen/PDMS) (Supelco, Bellefonte, PA, USA). The SPME fibre, conditioned for 30 min at 270 °C in the GC injector to eliminate residues, was exposed to the meat’s headspace for 30 min at 60 °C. Conditioning and exposure were carried out at 60 °C [30]. The VOCs were analysed using a single quadrupole GC/MS apparatus (TRACE GC/MS, Thermo-Finnigan, Waltham, MA, USA). The injector was set at 250 °C. For the first 3 min, the split was set in splitless mode, and the fibre was kept in the injector for 30 min. The GC system was coupled with a Varian CP-WAX-52 capillary column (60 m × 0.32 mm; coating thickness 0.5 μm), and the temperature program was set according to Povolo et al. [31] as follows: the oven temperature was held at 40 °C for 8 min, then programmed to 220 °C at a rate of 4 °C min^−1^ and held at 220 °C for 20 min. Helium was used as a carrier gas at a flow rate of 1.0 mL min^−1^. The transfer line and ion source were set at 250 °C. The filament emission current was 70 eV. A mass range from 35 to 270 mz was scanned at a rate of 1.6 amu s^−1^. The acquisition was carried out by electron impact using the full scan (TIC) mode. The VOCs quantification was performed at T0, T1, and T2.

The VOCs were identified by comparing the mass spectra of the Wiley library (version 2.0-11/2008), by injection of authentic standards (Sigma-Aldrich), and by calculating the LRI and matching them with bibliographical indexes [29,31]. The data were expressed as the peak percentage of total VOCs.

### 2.8. Evaluation of Calliphora Vomitoria Oviposition Deterrence on Beef Loaves

To prepare the beef loaves, we used the same minced beef as for the beef patties. Approximately 470 g (469.49 ± 1.94 g; mean weight ± SD, *n* = 12) of meat was weighed on the precision balance and manually shaped in prolate spheroidal loaves (height 28.02 ± 1.40 cm, length 12.60 ± 0.68 cm; mean measures ± SD, *n* = 12; surface ≅ 1109 cm^2^). A total of 12 beef loaves was prepared (three samples for each of the four treatments). The three untreated (control) loaves were individually wrapped in elastic, tubular cotton netting to maintain their shape and kept at room temperature for 60 min. The treated loaves were first wrapped in the netting, immersed for 30 s in the respective treatment (three in the 1.0% CH solution, three in the *L. nobilis* EO-enriched CH solution, and three in the *P. nigrum* EO-enriched CH solution), placed on a plastic mesh grill to allow the excess solution to drip off, and left to dry for 60 min. Using the precision balance, we determined that the weight of the CH or CH+EO covering was 28.22 ± 0.20 g (mean weight ± SD, *n* = 9). Therefore, we can assume that the amount of EO was 0.25 µL EO cm^−2^ of surface.

The rearing of *C. vomitoria* was maintained under laboratory conditions (23 °C, 60–70% RH, natural photoperiod 12/12 h day/night). In accord with the procedure used by Farina et al. [20], mature larvae were purchased from a commercial supplier of live fishing baits (Nonno Ippei, Vittoria Apuana, Italy) and kept in polypropylene boxes (27 × 21 × 12 cm) with a netted lid for ventilation until pupation. The pupae were then transferred into 75.0 × 75.0 × 115.0 cm knitted mesh and polyester tents (BugDorm-2400 Insect Rearing Tent, MegaView Science Co., Ltd., Taichung, Taiwan), which were provided with *ad libitum* water and a sucrose + yeast extract (4:1 *w*/*w*) solid diet for the emerging adults. Yeast extract was used as a protein source to support oviposition [32]. Species identification was carried out on twenty randomly selected adults using the specific identification keys [33].

The deterrence exerted by the proposed treatments against oviposition by *C. vomitoria* females was evaluated using the beef loaves randomly hung (wire length 12.0 cm) from the ceiling of a netted polytunnel (width 4.0 m, length 10.0 m, peak height 2.0 m). The polytunnel was located in the garden of the Entomology Laboratory of the Department of Agriculture, Food and Environment (University of Pisa, Pisa, Italy). For the trial, it was equipped with water sources and landing surfaces for the blowflies. The netted cover prevented the entry of other insects and the escape of our blowflies.

At T0, we released 150 *C. vomitoria* adults of undetermined sex (15–20 days old, sex ratio 1:1) inside the polytunnel to simulate a work environment where good manufacturing practices are not followed. After 48 h (T1), we collected all the egg masses laid on the loaves and weighed them on an analytical balance (KERN ABS-N, Kern & Sohn, Balingen, Germany). The seven blowflies found dead were removed and replaced to maintain a total of 150 specimens in the tunnel. All the loaves were then hung back in their original positions. The egg masses were collected and weighed again at T2 (after 96 h from the beginning of the trial). To calculate the total number of eggs laid, we determined the average weight of a single *C. vomitoria* egg.

The trial was conducted in March 2023, with a mean minimum temperature of 8.2 °C and a mean maximum temperature of 16.8 °C [34]. We selected this month to ensure that the mean daily temperature was above 13 °C, which is considered unsuitable for meat product preservation during most processing activities. The natural photoperiod at that time in Pisa was 12/12 h day/night.

### 2.9. Statistical Data Analyses

Data regarding the meat’s composition, colour parameters, TBARS level, VOCs from beef patties, and number of eggs laid by *C. vomitoria* on the beef loaves were analysed with the following linear model:

y_ijk_ = μ + C_i_ + T_j_ + C_i_ × T_j_ + ε_ijk_

where:

y_ijk_ = variable,
μ = mean,
C_i_ = fixed effect of the i^th^ treatment (control, CH, CH+ *L. nobilis* EO, CH+ *P. nigrum* EO),
T_j_ = fixed effect of the j^th^ time of storage (T0, T1, T2),
ε_ijk_ = random error.

For multiple comparisons of means, Tukey’s HSD post-hoc test (*p* < 0.05) was used. All analyses were performed using JMP Pro Software v 17.2 (SAS Institute Inc., Cary, NC, USA).

## 3. Results

### 3.1. Chemical Composition and Colour Parameters of Beef Patties

**Figure 1:**
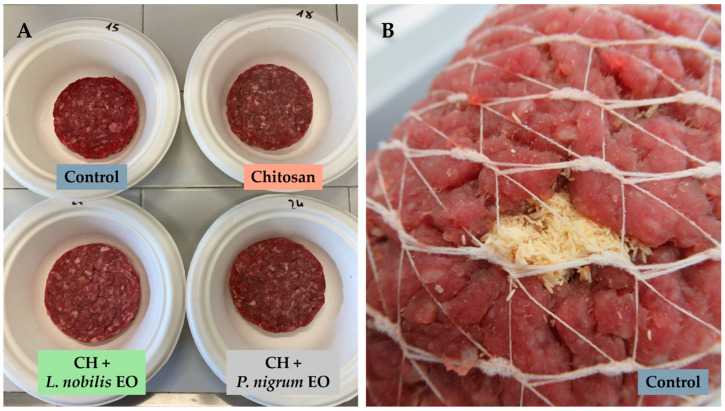
(**A**) Beef patties untreated (control) and treated with the chitosan (CH), *Laurus nobilis* essential oil (EO)-enriched CH, and *Piper nigrum* EO-enriched CH solutions after 96 h (T2) of storage at 4 °C. (**B**) An untreated (control) beef loaf with a cluster of eggs laid by *Calliphora vomitoria* after 48 h (T1) of exposure at around 13 °C in the netted polytunnel.

The chemical composition and colour parameters of the beef patties over the 4 days are reported in Table 1. No significant differences were observed in the chemical composition. Instead, all the treatments here proposed significantly affected, at least at a specific storage time (T0, T1, or T2), one of the CIE colour parameters recorded (L*, a*, and b*) or calculated (H* and C*). Some colour differences were visible to the naked eye, as shown in Figure 1A.

More specifically, lightness (L*) was significantly higher than in the control at all times with the three CH-containing treatments. The CH group showed the highest values (49.72, 50.19, and 49.32), closely followed by the *L. nobilis* EO-enriched CH (48.69, 49.96, and 47.64). In contrast, the untreated control recorded the lowest values (44.85, 46.37, and 44.03). Conversely, redness (a*) was significantly reduced by the treatments at all three times (from around 15 in the control to around 10 in the treatments), especially with the *P. nigrum* EO-enriched CH. Yellowness (b*) was also significantly reduced by the CH-containing treatments, but only after 96 h (decreases of 5.5, 15.5, and 13.5% from T0 to T2 in CH, CH + *L. nobilis* EO, and CH + *P. nigrum* EO, respectively). Consequently, the hue angle (H*) values calculated at all times and for all treatments were significantly higher than for the control (from around 47 in the control to around 54 in the treatments), and the chroma (C*) values were always significantly lower (from around 23 in the control to around 19 in the treatments) (Table 1).

### 3.2. Secondary Oxidation Products in Beef Patties

**Figure 2:**
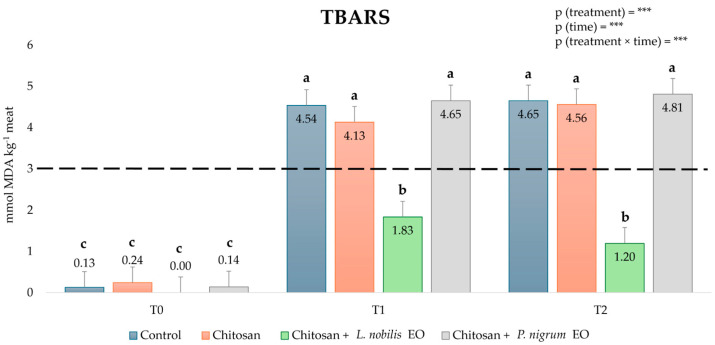
Effect of chitosan (CH) and *Laurus nobilis* or *Piper nigrum* essential oils (EOs)-enriched CH treatment on thiobarbituric acid-reacting substances (TBARS) level in beef. The dashed line indicates the minimum threshold for rancidity perception, according to Lanari et al. [35]. Means with different letters significantly differ for the treatment effect (*p* < 0.05). *** = *p* < 0.001. Times = T0 right after the treatment; T1 after 48 h; T2 after 96 h. Values are expressed in mmol of malonyldialdehyde (MDA) equivalent kg^−1^ meat.

The presence of secondary oxidation products, evaluated through the TBARS test, revealed significant differences depending on the treatment and storage time (T0, T1, or T2), as represented in Figure 2. Immediately after the treatments, the TBARS level was, as expected, low (around 0.1 mmol MDA kg^−1^ meat) and comparable among all the samples. As time passed, the values increased in all samples and exceeded the minimum threshold for the rancidity perception, set at 3 mmol MDA kg^−1^ meat [35], after just 2 days of storage in the control, CH, and *P. nigrum* EO-enriched CH patties. The only treatment able to keep the TBARS well below the threshold (1.83 and 1.20 mmol MDA kg^−1^ meat after 48 and 96 h, respectively) was the *L. nobilis* EO-enriched CH solution.

### 3.3. Volatile Organic Compounds (VOCs) Released from Beef Patties

The SPME-GC/MS analysis of beef patties revealed the total presence of 24 compounds (Table S1) across all treatments and storage times (T0, T1, and T2). Specifically, the untreated control was characterised by typical compounds of meat products such as acids (hexanoic acid and pentanoic acid), alcohols (ethanol, 1-pentanol, 2-ethyl-1-hexanol, 2-hexen-1-ol, and 3-methyl-1-pentanol), aldehydes (2-ethylbutanal and 2,3,6-trichlorobenzaldehyde), esters (ethyl acetate), hydrocarbons (1,3-dimethylbenzene), ketones (2-butanone and 3-hydroxy-2-butanone), and others, such as 2,4-bis(1-methylethyl)-phenol. The VOC profiles of the CH-treated samples were similar to those of the controls, with slight differences in the quantities but not the types of compounds detected. Specifically, some statistically significant differences were observed in 2-hexen-1-ol (higher in CH at T0), hexanoic acid (higher in CH at T2), 2-ethyl-1-hexanol (higher in control at T1), and pentanoic acid, 2-butanone, and ethyl acetate (all higher in control at T1 and T2). Interestingly, the fatty acid ester ethyl octanoate was detected exclusively in the control and plain CH samples and significantly increased from T0 to T2 (by 138.37 and 306.98%, respectively). Ethanol was the most abundant compound, especially after 4 days (from 3.05 at T0 to 26.44 at T2 in the control and from 0.78 to 21.71 when applying CH). Traces of typical aromatic hydrocarbons, phenols, monoterpenes, and monoterpenoids of EOs were also revealed, probably due to contamination.

In the *L. nobilis* EO-enriched CH-treated samples, significant amounts of 1,8-cineole (from 302.31 at T0 to 184.06 at T2), linalool (from 200.13 to 100.97), p-menthene (from 108.02 to 85.25), and m-cymene (from 98.06 to 20.79), together with smaller amounts of β-phellandrene, 1-pentanol, and γ-terpinene were found. The compound 1,4-hexadiene was found only in the formulation containing the *L. nobilis* EO. Regarding the *P. nigrum* EO-enriched CH-treated beef, we noticed a substantial presence of p-menthene (from 678.76 at T0 to 210.44 at T2), followed by δ-2-carene (from 297.49 to 93.76), γ-terpinene (from 98.13 to 21.31), β-phellandrene (from 79.14 to 22.46), and smaller amounts of linalool.

For both the EOs, a reduction in the abundance of specific compounds was observed over time (from T0 to T2). For instance, over 4 days, γ-terpinene decreased by 83.48 and 78.27% in the *L. nobilis* and *P. nigrum* CH formulations, β-phellandrene by 82.31 and 71.62%, m-cymene by 78.80 and 76.75%, linalool by 49.55 and 79.95%, 1,8-cineole in the former by 39.12%, and δ-2-carene and p-menthene in the latter by 68.49 and 69.00%, respectively.

### 3.4. Oviposition Deterrence Exerted on Calliphora vomitoria

The number of eggs laid on all the beef loaves was determined by calculating the mean weight of a single egg, which corresponds to 0.146 ± 0.04 mg (mean weight ± SD, *n* = 6). The total number of eggs laid on all the samples at both times was 13,983.

All three CH-containing treatments were effective in deterring the oviposition of *C. vomitoria* females on the beef loaves hung from the ceiling of the netted polytunnel. Specifically, at T1 (after 48 h), the protection provided by the two EOs-enriched CH solutions was complete (no eggs were found on the beef loaves), and plain CH reduced oviposition by 88.32% compared to the untreated control (Figure 1B). At T2 (after 96 h), the *P. nigrum* EO-enriched CH solution still provided nearly complete protection (99.75%), followed by the *L. nobilis* EO-enriched CH solution (88.83%) and plain CH (66.22%). Statistically significant differences were found between the three CH-containing treatments and the untreated control (*p* = 0.0005), but there were no significant differences among the treatments themselves nor between T1 and T2.

## 4. Discussion

The gas chromatography/electron impact-mass spectrometry analysis of the EOs involved in this study is fully reported in Farina et al. [20]. Briefly, in the *L. nobilis* EO, the oxygenated monoterpenes 1,8-cineole (28.1%) and α-terpinyl acetate (17.5%) were the most abundant components. In the *P. nigrum* EO, over 60% of the composition was characterised by sesquiterpene hydrocarbons, including 45.7% of β-caryophyllene. The chemical compositions of both EOs align with those reported in the literature by other authors. Similarly to our accession, laurel EOs extracted from plants collected in Greece and Georgia contained 1,8-cineole (30.8 and 29.2%, respectively) and α-terpinyl acetate (14.9 and 22.6%, respectively) as major components. In the Georgian EO, 8.1% of methyl eugenol was also found (7.3% in our sample) [36]. Regarding black pepper EOs, β-caryophyllene is often reported as their major component. Indeed, this sesquiterpene constituted 51.12% of the composition of a sample used by Andriana et al. [37] and 47.14–50.88% of one analysed by Rmili et al. [38].

Both EOs here employed have been evaluated before by a group of expert sensory analysts [20]. On a 0 to 9 scale, the *L. nobilis* and *P. nigrum* EOs simply diluted in ethanol received scores of 8.31 and 8.71, respectively, for their overall pleasantness. The former was associated with fresh and resinous notes while the latter was characterised by citrusy and floral scents. When added to the CH solution and applied on beef at 0.23 µL EO cm^−2^ of treated surface (i.e., the same amount we used on our patties), the overall pleasantness was 7.52 for the *L. nobilis* EO and 7.11 for the *P. nigrum* EO [20].

The colour parameters of beef patties, which are crucial cues used by consumers to judge meat freshness through its appearance [39], were affected by our treatments and storage time. All the CH coatings resulted in a significant increase in lightness, which was more pronounced in the CH (+10.72%) and *L. nobilis* EO-CH enriched (+7.58%) groups. This phenomenon is in accordance with Farina et al. [20], which reported higher L* values in beef patties coated with different CH solutions after 4 and 7 days of storage at 4 °C. Furthermore, Jo et al. [40] observed the same effect when adding CH oligomers to cooked pork sausage, Giatrakou et al. [41] when applying CH-thyme EO treatments on a ready-to-cook poultry preparation, and Petrou et al. [42] when dipping chicken breast fillets in CH and oregano EO-enriched CH. Therefore, CH itself likely creates a coating that increases the reflection of light, making the meat look brighter.

A decreasing trend in a*, b*, and C* values was observed from 0 to 4 days of storage in our study, together with a consequent H* increase. Overall, these changes can be attributed to the gradual oxidation of myoglobin and the accumulation of metmyoglobin over time [43]. Lower a* values were reported in pork patties containing rosemary compared to those with sage or soy proteins after 6 days of storage at 4 °C, despite the overall better effect of rosemary against lipid oxidation compared to the other natural additives [44]. Other studies reported an increase in redness and yellowness [40] or the absence of colour changes attributable to CH [42]. These discrepancies, in addition to pigment oxidation, might be due to the different meat types (e.g., beef, chicken, pork, lamb) and cut/preparation (e.g., steak, fillet, patty, sausage) used.

The presence of secondary oxidation products increased over time in all the samples, but the *L. nobilis* EO-enriched CH solution was able to maintain levels well below the minimum threshold for the rancidity perception [35]. Chitosan is already used by the food industry for its antioxidant properties [14], but according to our results, it appears that a specific EO with a positive synergy is required to actively protect the meat. For instance, a similar effect was observed when using CH coatings with rosemary or oregano EOs on beef steaks [45], chicken breasts [42], and chicken burgers [46], or active films based on CH and rosemary EO on fresh poultry meat [47], as well as CH with a Brazilian EO rich in 1,8-cineole on ground beef [48]. Even a mixture of CH and a mint extract incorporated into the recipe of commercially produced pork cocktail salami made them more resistant to lipid oxidation throughout the storage period [49].

The acids, alcohols, aldehydes, esters, hydrocarbons, and ketones that make up the VOCs released by the untreated beef samples have all been described before by other authors working on meat characterisation through SPME-GC/MS or other techniques [50]. As odorant molecules, hexanoic acid, 1-pentanol, and 3-hydroxy-2-butanone are described as fatty, pentanoic acid as meaty, 2,4-bis(1-methylethyl)-phenol as woody, pentanol as peanut, 2-butanone as green, ethanol and 1,3-dimethylbenzene as sweet, and ethyl acetate and 3-methyl-1-pentanol as fruity [51,52,53,54]. Surprisingly, treatment with the CH solution only slightly altered the volatile emissions, suggesting a mild effect on the patties’ smell.

The presence of specific plant secondary metabolites among the VOCs deriving from the EO-containing beef samples was consistent with the chemical compositions of the EOs themselves. As mentioned earlier, 1,8-cineole (28.1%) was the main compound found in the *L. nobilis* EO, with smaller amounts of linalool (5.5%) and γ-terpinene (0.6%) also detected. Different isomers of other constituents were also present, such as m-cymene (0.5% of p-cymene in the EO), β-phellandrene (0.2% of α-phellandrene), and δ-2-carene (0.1% of δ-3-carene). Eugenyl acetate was likely derived from eugenol (3.4% in the EO) via acetylation and p-menthene from the rearrangement of terpenes or cyclic compounds. The same phenomenon could explain the presence of p-menthene among the VOCs emitted by the beef treated with the *P. nigrum* EO-enriched CH solution. Even in this case, compounds corresponding to the original EO composition (i.e., linalool = 0.3% in the EO) or their isomers (e.g., δ-2-carene from δ-3-carene = 4.7%, β-phellandrene from α-phellandrene = 0.6%, γ-terpinene from δ-terpinene = 0.3%, m-cymene from p-cymene = 0.2%) were identified.

As odorant molecules, 1,8-cineole is described as fresh and eucalyptus-like, δ-2-carene as sweet, linalool as floral and citrusy, p-menthene as minty, β-phellandrene as peppery-minty, and γ-terpinene as green and citrusy [55]. In all the beef patties treated with the EO-containing formulations, a decrease in the prevalence of these secondary metabolites was observed over time. This lack of persistence can be attributed to their volatility, which is related to their low molecular weight and is one of the main limitations to the use of EOs and their individual components in applications that require long-term release of the active ingredient [56].

Ethanol was detected in all beef patties and has been negatively correlated with changes in sensory properties [57] and spoilage [58] in previous studies. Indeed, its trend during the storage period followed the same pattern as TBARS. The quantity of ethanol identified through SPME-GC/MS analysis in all the samples at T0 was comparable. It then increased at T1 and T2, with only the *L. nobilis* EO-enriched CH solution being able to maintain the ethanol level statistically lower than those of the other treatments and control. Both the EO-containing samples also showed higher levels of hexanoic acid and 1-pentanol (associated with fatty odours), the *P. nigrum* EO increased 2-butanone (green odour) and 1,3-dimethylbenzene (sweet odour), and the *L. nobilis* EO intensified the release of 2,4-bis(1-methylethyl)-phenol (woody odour) [55].

Regarding the protection against *C. vomitoria* oviposition, our findings are in line with the evidence reported by Farina et al. [20]. In their oviposition deterrence tests (conducted under laboratory conditions for 24 h with the same blowfly *C. vomitoria* as the target pest), they found that the treatment of beef with their *P. nigrum* EO-enriched CH solution (at 0.23 µL EO cm^−2^ of treated surface) was the most effective. Treatment with an ethanolic solution of the same *P. nigrum* EO was also promising, but as already mentioned, EOs are volatile, and their bioactivity is better preserved when added to a stabilising matrix such as CH. Our EO-CH formulations (at a similar amount of 0.25 µL EO cm^−2^) were able to significantly protect beef from blowfly oviposition for up to 96 h, confirming the stabilising effect of CH on the EOs’ composition. Compounds such as δ-2-carene, 1,8-cineole, m-cymene, linalool, p-menthene, β-phellandrene, and γ-terpinene were likely responsible for this deterrent activity, as they were the most abundant plant secondary metabolites among the VOCs released by the EO-containing beef samples.

Concerning the use of other EOs as deterrents against *C. vomitoria* blowflies, several studies have been conducted involving EOs extracted from aromatic plants used as culinary herbs and spices, but always unformulated (simply diluted in ethanol). For instance, a tarragon EO at 0.05 µL EO cm^−2^ completely (100%) protected beef for 24 h [59]. At 0.32 µL EO cm^−2^, Bedini et al. [60] achieved 90% protection of pork for 24 h using a thymol/γ-terpinene chemotype oregano EO. Bedini et al. [61] also reported complete protection of pork for 24 h with a garlic EO starting from 1.25 µL EO cm^−2^ and nearly 70% protection for 72 h, but with a higher amount of 5.0 µL EO cm^−2^. These studies suggest that, even if such EOs are effective deterrents against *C. vomitoria*, high concentrations are necessary to obtain substantial protection of meat products over a beneficial period.

Building upon this, there have been promising attempts to formulate EOs or their chemical components in liquid, film, or nanoparticle CH to enhance and prolong their efficacy against insect pests [19]. Among the deterrent solutions proposed, we can list liquid CH enriched with a mandarin EO to protect kumquat fruits from the oviposition of *Ceratitis capitata* (Diptera: Tephritidae) for up to 96 h [23] or with bay and black pepper EOs to shield ham and cheese from *Piophila casei* (Diptera: Piophilidae) over the same period of time [62]. Furthermore, a CH coating with a field ferulago EO applied to common beans hindered the oviposition by *Acanthoscelides obtectus* (Coleoptera: Chrysomelidae) for 14 days [63]. As a repellent, a bitter orange EO-CH film had a long-lasting effect on *Sitophilus oryzae* (Coleoptera: Dryophtoridae) for up to 28 days [64].

## 5. Conclusions

The use of wrapping films to reduce the loss and waste of meat products is one of the viable solutions available to the food industry. However, to meet the growing consumer demand for healthier, safer, and more eco-friendly alternatives, research has shifted towards natural compounds with innate film-forming, antimicrobial, and antioxidant properties, such as CH and EOs.

The *L. nobilis* EO-enriched CH solution here tested increased the meat colour’s lightness compared to the control, while keeping redness and yellowness comparable to it. The treatment also demonstrated remarkable antioxidant activity, as evidenced by the lower TBARS values and reduced ethanol content among the VOCs for at least 96 h. Even if 4 days are not enough to permanently assure the beneficial effects on colour and lipid oxidation, the preliminary results here obtained are encouraging. The *L. nobilis* EO-enriched CH solution also enhanced the perception of fatty and woody notes in the meat, together with the fresh, green, and citrusy aromas related to the specific EO, and provided significant protection against *C. vomitoria* oviposition for 96 h compared to the control. This dual defence against some abiotic and biotic factors affecting meat spoilage was scarcely investigated before, and our promising results could pique the interest of the food industry towards the natural compounds CH and EOs.

For easier application, we suggest developing a liquid spray formulation specifically reinforced with co-formulants and/or obtained through micro-/nanotechnologies. This improvement would further stabilise and prolong the effects of the volatile composition of the EO [65]. Alternatively, advanced multilayer mini-/nanoemulsions [66] and robust films [67] of CH with other biopolymers and incorporating EOs are currently under development.

Furthermore, it would be interesting to investigate the antimicrobial efficacy of our CH-EO formulation against the specific meat spoilage microorganisms [5,18], possibly including those carried by blowflies. Ultimately, although the *L. nobilis* EO has been previously selected by expert panellists for its favourable odour compatibility with meat, a sensory evaluation of the treated beef after cooking using appropriate methods would help confirm the overall acceptability of the final product.

## Figures and Tables

**Table 1:** Effect of chitosan (CH) and *Laurus nobilis* or *Piper nigrum* essential oils (EOs)-enriched CH treatment on chemical composition (g 100 g^−1^ meat) and colour parameters (according to the Commission Internationale de l’Éclairage, CIE) of beef.

	Control			Chitosan			CH+ *L. nobilis* EO			CH+ *P. nigrum* EO			SE	*p*-Value		
	T0	T1	T2	T0	T1	T2	T0	T1	T2	T0	T1	T2		Treat	Time	Treat × Time
**Moisture**	72.46	73.47	72.13	73.82	74.29	73.42	72.79	74.06	72.74	71.62	73.38	73.81	1.56	ns	ns	ns
**Protein**	22.77	24.47	22.13	21.76	21.60	21.91	23.93	22.08	21.72	22.32	21.44	22.64	0.85	ns	ns	ns
**Lipid**	2.66	2.38	2.02	1.87	1.72	1.92	2.82	2.07	1.83	2.21	1.54	2.53	0.15	ns	ns	ns
**Ash**	1.15	1.17	1.13	1.17	1.22	1.10	1.08	1.09	1.03	1.11	1.14	1.13	0.07	ns	ns	ns
L*	44.85 ^β^	46.37 ^β^	44.03 ^β^	49.72 ^α^	50.19 ^α^	49.32 ^α^	48.69 ^α^	49.96 ^α^	47.64 ^α^	46.52 ^β^	47.28 ^β^	48.71 ^β^	1.76	*	ns	ns
a*	17.67 ^α^	15.40 ^α^	14.89 ^α^	11.66 ^β^	10.50 ^β^	10.81 ^β^	13.90 ^β^	12.98 ^β^	11.62 ^β^	12.22 ^β^	11.34 ^β^	7.54 ^β^	0.67	***	***	ns
b*	17.71 ^a^	16.88 ^a^	16.75 ^a^	15.60 ^a^	15.57 ^a^	14.74 ^b^	17.38 ^a^	16.55 ^a^	14.69 ^b^	15.44 ^a^	15.66 ^a^	13.35 ^b^	0.54	***	***	*
H*	45.14 ^β^	47.75 ^β^	48.41 ^β^	53.74 ^α^	56.23 ^α^	53.48 ^α^	51.54 ^α^	51.92 ^α^	51.71 ^α^	51.67 ^α^	54.21 ^α^	60.59 ^α^	1.51	***	*	*
C*	25.03 ^α^	22.90 ^α^	22.45 ^α^	19.54 ^β^	18.82 ^β^	18.33 ^β^	22.29 ^β^	21.08 ^β^	18.79 ^β^	19.70 ^β^	19.41 ^β^	15.38 ^β^	0.69	***	***	ns

L* = lightness; a* = redness; b* = yellowness; H* = hue angle; C* = chroma. Means with different Latin letters significantly differ for the treatment × time effect (*p* < 0.05); means with different Greek letters significantly differ for the treatment effect (*p* < 0.05). Treat = treatment. Times = T0 right after the treatment; T1 after 48 h; T2 after 96 h. SE = standard error. * = *p* < 0.05; *** *p* < 0.001; ns = not significant.

## Data Availability

The original contributions presented in this study are included in the article/Supplementary Materials. Further inquiries can be directed to the corresponding author.

## Supplementary Materials

The following supporting information can be downloaded at: https://www.mdpi.com/article/10.3390/foods14050897/s1, Table S1: Effect of chitosan (CH) and *Laurus nobilis* or *Piper nigrum* essential oils (EOs)-enriched CH treatment on volatile organic compounds (VOCs) released by beef meat.

## Abbreviations

The following abbreviations are used in this manuscript:

CH	Chitosan
EO	Chitosan
MDA	Malonyldialdehyde
PVC	Polyvinylidene chlorid
SPME-GC/MS	Solid phase microextraction-gas chromatography/mass spectrometry
TBA	Thiobarbituric acid
TBARS	Thiobarbituric acid-reacting substances
VOC	Volatile organic compound
