# Supplementary material for: Benefits of Essential Oil-Enriched Chitosan on Beef: From Appearance and Odour Improvement to Protection Against Blowfly Oviposition
Source: Foods. 2025 Mar 6;14(5):897. doi: 10.3390/foods14050897 (PMC11898430; doi:10.3390/foods14050897)
Supplement: Supplementary file 1 [file foods-14-00897-s001.zip › foods-3494061-supplementary.pdf]

Table S1. Effect of chitosan (CH) and Laurus nobilis or Piper nigrum essential oils (EOs)-enriched CH treatment on volatile organic compounds (VOCs) released by beef meat.

Compounds	Control			Chitosan			CH + *L. nobilis* EO			CH + *P. nigrum* EO			SE	*p*-Value		
	T0	T1	T2	T0	T1	T2	T0	T1	T2	T0	T1	T2		Treat	Time	Treat x Time
2,3,6-trichlorobenzaldehyde	6.39 ^{a}	4.12 ^{b}	2.98 ^{b}	6.54 ^{a}	2.34 ^{b}	2.91 ^{b}	8.04 ^{a}	6.00 ^{a}	6.00 ^{a}	8.23 ^{a}	7.71 ^{a}	1.64 ^{b}	0.73	***	***	***
ethyl acetate	- ^{d}	0.95 ^{b}	1.18 ^{a}	- ^{d}	0.39 ^{c}	0.91 ^{b}	- ^{d}	1.24 ^{a}	0.67 ^{b}	- ^{d}	1.58 ^{a}	0.75 ^{b}	0.12	*	***	***
2-butanone	2.12 ^{a}	1.48 ^{b}	0.72 ^{c}	1.18 ^{b}	0.53 ^{c}	0.76 ^{c}	1.66 ^{b}	1.33 ^{b}	0.85 ^{c}	2.13 ^{a}	1.92 ^{a}	1.44 ^{b}	0.31	***	***	***
ethanol	3.05 ^{e}	15.28 ^{c}	26.44 ^{a}	0.78 ^{e}	5.60 ^{d}	21.71 ^{b}	- ^{e}	7.82 ^{d}	11.71 ^{c}	1.80 ^{e}	18.61 ^{b}	24.82 ^{a}	1.77	***	***	**
γ-terpinene	0.25 ^{e}	0.39 ^{e}	0.70 ^{e}	0.57 ^{e}	0.57 ^{e}	0.78 ^{e}	46.66 ^{b}	18.60 ^{c}	7.71 ^{d}	98.13 ^{a}	48.68 ^{b}	21.31 ^{c}	4.48	***	***	***
β-phellandrene	- ^{f}	0.72 ^{f}	0.86 ^{f}	0.15 ^{f}	0.47 ^{f}	0.87 ^{f}	29.91 ^{c}	14.70 ^{d}	5.29 ^{e}	79.14 ^{a}	42.10 ^{b}	22.46 ^{c}	3.16	***	***	***
1,3-dimethylbenzene	0.83 ^{b}	0.47 ^{b}	1.28 ^{b}	0.39 ^{b}	0.69 ^{b}	1.21 ^{b}	1.29 ^{b}	1.56 ^{b}	2.57 ^{b}	64.44 ^{a}	68.16 ^{a}	65.84 ^{a}	6.74	***	**	***
δ-2-carene	- ^{e}	2.77 ^{e}	5.05 ^{e}	- ^{e}	2.63 ^{e}	5.03 ^{e}	46.14 ^{d}	26.18 ^{d}	10.18 ^{d}	297.49 ^{a}	176.47 ^{b}	93.76 ^{c}	11.30	***	***	***
1,8-cineole	- ^{c}	1.70 ^{c}	1.31 ^{c}	0.58 ^{c}	1.65 ^{c}	1.60 ^{c}	302.31 ^{a}	220.02 ^{b}	184.06 ^{b}	- ^{c}	1.71 ^{c}	1.32 ^{c}	20.14	***	***	***
ethyl octanoate	0.86 ^{c}	0.66 ^{c}	2.05 ^{a}	0.43 ^{c}	0.50 ^{c}	1.75 ^{b}	- ^{d}	- ^{d}	- ^{d}	- ^{d}	- ^{d}	- ^{d}	0.22	***	***	**
1-pentanol	0.85 ^{d}	1.33 ^{d}	1.09 ^{d}	0.84 ^{d}	1.01 ^{d}	1.69 ^{d}	44.94 ^{a}	19.53 ^{b}	7.06 ^{c}	13.58 ^{b}	8.46 ^{c}	6.25 ^{c}	2.99	***	***	***
m-cymene	- ^{d}	1.70 ^{d}	2.80 ^{d}	0.15 ^{d}	1.23 ^{d}	2.84 ^{d}	98.06 ^{a}	41.66 ^{b}	20.79 ^{c}	26.50 ^{c}	17.99 ^{c}	6.16 ^{d}	5.99	***	***	***
3-hydroxy-2-butanone	0.64 ^{b}	1.07 ^{a}	1.26 ^{a}	0.75 ^{b}	0.80 ^{b}	1.19 ^{a}	1.13 ^{a}	1.10 ^{a}	1.24 ^{a}	1.02 ^{a}	1.72 ^{a}	1.72 ^{a}	0.53	***	***	***
2-ethylbutanal	1.04 ^{c}	1.10 ^{c}	1.35 ^{c}	0.49 ^{d}	0.93 ^{c}	2.29 ^{b}	1.67 ^{c}	2.39 ^{b}	1.65 ^{c}	1.25 ^{c}	3.32 ^{a}	2.00 ^{b}	0.22	***	***	***
3-methyl-1-pentanol	0.71 ^{c}	0.34 ^{d}	0.85 ^{c}	0.33 ^{d}	0.35 ^{d}	0.87 ^{c}	1.60 ^{a}	0.88 ^{c}	1.04 ^{b}	0.23 ^{d}	0.36 ^{d}	1.23 ^{b}	0.14	***	*	*
1,4-hexadiene	- ^{c}	- ^{c}	- ^{c}	- ^{c}	- ^{c}	- ^{c}	3.94 ^{a}	3.90 ^{a}	2.02 ^{b}	- ^{c}	- ^{c}	- ^{c}	0.21	***	***	***
2-hexen-1-ol	0.91 ^{a}	2.61 ^{b}	3.67 ^{a}	1.58 ^{c}	2.27 ^{b}	3.61 ^{a}	1.09 ^{c}	1.86 ^{a}	1.57 ^{a}	1.68 ^{c}	1.34 ^{c}	1.12 ^{c}	0.43	**	*	***
2-ethyl-1-hexanol	1.43 ^{b}	2.91 ^{a}	2.72 ^{a}	1.53 ^{b}	1.66 ^{b}	3.28 ^{a}	4.42 ^{a}	3.80 ^{a}	3.95 ^{a}	3.98 ^{a}	2.35 ^{a}	0.40 ^{c}	1.43	***	***	***
linalool	0.51 ^{e}	2.98 ^{e}	4.08 ^{e}	0.59 ^{e}	1.47 ^{e}	9.61 ^{e}	200.13 ^{a}	165.78 ^{b}	100.97 ^{c}	37.24 ^{d}	33.32 ^{d}	7.47 ^{e}	11.45	***	**	**

p-menthene	1.35 c	1.63 c	4.02 c	0.60 c	1.85 c	5.08 c	108.02 b	122.61 b	85.25 b	678.76 a	393.01 b	210.44 b	43.90	***	**	***
pentanoic acid	0.97 b	1.96 a	2.34 a	0.78 b	0.84 b	0.45 b	1.93 a	1.50 b	1.45 b	1.40 b	1.54 b	1.85 a	0.33	***	***	***
hexanoic acid	1.55 c	3.59 c	3.31 c	3.07 c	3.90 c	5.91 a	7.60 a	9.41 a	6.12 a	8.61 a	8.03 a	4.85 b	1.06	***	***	***
2,4-bis(1-methylethyl)-phenol	1.89 c	1.78 c	1.32 c	1.85 c	1.70 c	5.66 bc	280.46 a	233.21 a	99.80 b	28.44 b	9.68 bc	6.63 bc	15.30	***	***	***
eugenyl acetate	1.80	3.14	3.80	-	1.46	3.41	15.33	13.85	13.54	17.02	24.18	12.48	5.04	***	*	ns

Means within the same variables with different letters significantly differ for the time effect ($p < 0.05$). - = not detected. Treat = treatment. Times = T0 right after the treatment; T1 after 48 hours; T2 after 96 hours. SE = Standard error. * = $p < 0.05$; ** = $p < 0.01$; *** $p < 0.001$; ns = not significant.
